# Selective Inhibition of *Yersinia enterocolitica* Type III Secretion by *Lindera obtusiloba* Extract and Cinnamtannin B1

**DOI:** 10.3390/pharmaceutics17091217

**Published:** 2025-09-18

**Authors:** Jin-Hee Yoo, Tae-Jong Kim

**Affiliations:** Department of Forest Products and Biotechnology, Kookmin University, 77 Jeongneungro, Seongbukgu, Seoul 02707, Republic of Korea; jhy0730@kookmin.ac.kr

**Keywords:** *Yersinia enterocolitica*, type III secretion system, virulence factor inhibition, cinnamtannin B1, *Lindera obtusiloba*, macrophage protection

## Abstract

**Background/Objectives**: Selective inhibition of bacterial virulence factors is a promising strategy to convert pathogenic bacteria into non-pathogenic commensals, circumventing the challenge of antibiotic resistance. This approach enables the host immune system to eliminate virulence-attenuated pathogens. **Methods**: In this study, we evaluated the effects of *Lindera obtusiloba* Blume extract and cinnamtannin B1, the active component of the ethyl acetate fraction, on the type III secretion system (T3SS) of *Yersinia enterocolitica*. **Results**: The ethyl acetate fraction, at 100 mg/L, effectively suppressed all three T3SS components—the flagellar, Ysa, and Ysc T3SSs. Cinnamtannin B1, isolated from the ethyl acetate fraction through separation and identified through nuclear magnetic resonance spectrometer analysis, significantly inhibited flagellar and Ysa T3SS secretion, while selectively inhibiting expression of key effector proteins YopH and YopO in the Ysc T3SS. Additionally, cinnamtannin B1 reduced *Y. enterocolitica*-induced RAW 264.7 macrophage mortality and prevented poly (ADP-ribose) polymerase degradation, a marker of apoptosis. **Conclusions**: These findings suggest cinnamtannin B1 from *L. obtusiloba* as a selective T3SS-targeting compound with mechanistic potential for anti-virulence intervention. Further in vivo validation will be necessary to evaluate its therapeutic applicability.

## 1. Introduction

Traditional antibiotics target bacterial-specific metabolisms to selectively kill bacteria. However, this creates selective pressure that accelerates the emergence of antibiotic-resistant strains [1]. In contrast, inhibiting bacterial virulence factors offers a novel approach to infection control by disabling pathogenesis rather than killing bacteria [2]. This strategy minimizes selective pressure, preserves beneficial microbiota, and reduces the development of resistance [3]. By inhibiting virulence factors, pathogenic bacteria can be made non-pathogenic, allowing the host immune system to clear infections.

The type III secretion system (T3SS) is a critical virulence factor in many Gram-negative bacteria, including *Yersinia* spp., *Shigella* spp., *Pseudomonas aeruginosa*, enteropathogenic *Escherichia coli*, enterohemorrhagic *E. coli*, and *Chlamydia* spp., acting as a molecular syringe that delivers effector proteins directly into host cells [4], where they interfere with immune functions. Because of its essential role in pathogenesis, the T3SS is a promising target for anti-virulence therapeutic development [5].

*Yersinia enterocolitica* is a foodborne pathogen responsible for gastrointestinal infections and systemic diseases in humans [6]. Its ability to cause infection depends significantly on the T3SS, making it an excellent model organism for studying the T3SS as a virulence factor. Furthermore, the increasing prevalence of antibiotic-resistant *Y. enterocolitica* strains demands alternative therapeutic strategies [7].

*Yersinia enterocolitica* possesses three distinct T3SSs: the flagellar T3SS, essential for motility and host invasion via the export of Fops (flagellar outer proteins) [8]; the Ysa T3SS, involved in the secretion of Ysps (*Yersinia* secreted proteins) and early-stage host interactions [9]; and the Ysc T3SS, which facilitates the delivery of Yops (*Yersinia* outer proteins), key effector proteins critical for disrupting host immune responses [10]. The three T3SSs are both specific and complementary, ensuring the survival of bacteria in the environment and in their hosts [11].

The genes encoding the Ysc T3SS are located on a ~70 kb plasmid, the pYV plasmid. Yop effectors play a pivotal role in immune evasion by suppressing macrophage and polymorphonuclear leukocyte phagocytosis, inhibiting the activation of T and B lymphocytes, and modulating cytokine production by T and B lymphocytes [12]. The secretion of Yops via the Ysc T3SS occurs even in the absence of direct host cell contact under specific environmental conditions, e.g., 37 °C without calcium [13,14].

The Ysa T3SS is encoded by the ysa locus in the chromosome of *Y. enterocolitica* [9]. During the earliest phase of infection, the Ysa T3SS contributes to *Y. enterocolitica* colonization of gastrointestinal tissues [15]. Ysa T3SS-defective mutants may exhibit reduced virulence in mice after oral administration [16]. The in vitro secretion of Ysp by the Ysa T3SS can be induced by growing *Y. enterocolitica* at 26 °C in a high salt (0.49 M NaCl) environment [17].

The secretion-dependent phospholipase activity of YplA, a phospholipase A in *Y. enterocolitica* that contributes pathogenicity and infectivity [18], can be used to assess the functionality of the T3SS [12,19]. The quantification of YplA activity allows the assessment of T3SS function and provides a powerful platform for the identification and characterization of T3SS inhibitors.

Building on prior research into the antimicrobial and antibiofilm activities of plant-derived extracts [20,21], *Lindera obtusiloba* Blume, commonly known as the blunt-leaved spicebush, is a deciduous shrub native to East Asia, with its range covering parts of Korea, Japan, and China. It is commonly found in forested and mountainous regions, where it contributes to the local ecosystem. Beyond its ecological importance, the plant has been used as an herbal medicine. In traditional medicine, various parts of *L. obtusiloba*—including its leaves, bark, and roots—have been used to treat a range of ailments, such as inflammation [22], liver disorders [23], and pain [24]. Its pharmacological potential is attributed to a diverse array of bioactive compounds, including flavonoids, tannins, alkaloids, and lignans [25]. These compounds are known for their antioxidant [25], anti-inflammatory [22], antimicrobial [26], and hepatoprotective [27] effects, making *L. obtusiloba* a rich source of natural therapeutic agents.

Cinnamtannin B1 (PubChem Identifier: CID 475277), a condensed tannin derived from natural sources, including *L. obtusiloba* [25], has diverse biological activities, such as antioxidant properties [28,29], and potential medical applications in antiviral [30] and antitumor [31,32] treatments. It has also been shown to modulate key cellular pathways, including those involved in inflammation [33]. Its ability to interact with proteins and enzymes also suggests potential for use in the therapeutic modulation of biological systems.

The purpose of this study is to identify and characterize compounds that selectively inhibit the T3SS in *Y. enterocolitica*, focusing on cinnamtannin B1 from *L. obtusiloba*. Unlike other polyphenolic T3SS inhibitors reported to date, cinnamtannin B1 appears to selectively inhibit the expression and secretion of YopH and Yop*O*—key effectors in immune evasion—rather than exerting broad inhibition. This study aims to evaluate the anti-virulence potential of cinnamtannin B1 and its role in suppressing T3SS-mediated pathogenesis in *Y. enterocolitica*, for future translational development studies in the context of antibiotic resistance.

## 2. Materials and Methods

### 2.1. Bacterial and Culture Media

*Yersinia enterocolitica* KT0001 (KCCM 41657), a plasmid-cured strain lacking the pYV virulence plasmid, was purchased from the Korean Culture Center of Microorganisms (Seoul, Republic of Korea). *Y. enterocolitica* KT0002, which possesses resistance to nalidixic acid, was derived from KCCM 41657 via spontaneous mutation [34]. Nalidixic acid resistance was required to facilitate the selection of transconjugants during the introduction of pYV100 [19], a derivative of the wild-type pYV virulence plasmid [9], via triparental mating. The resulting strain, KT0003, thus harbors pYV100 [34]. To enable screening based on phospholipase activity, pGY100—a YplA overexpression plasmid [12]—was introduced into KT0003 by conjugation, resulting in strain KT0004. Notably, KT0004 was used exclusively in experiments requiring quantification of phospholipase activity. The RAW 264.7 mouse macrophage cell line (ATCC TIB-71) was obtained from the Korean Cell Line Bank (Seoul, Republic of Korea).

*Yersinia enterocolitica* strains were grown at 26 °C in TYE medium (1% tryptone, 0.5% yeast extract) and on 1.5% agar TYE media plates. The strains were cultured in TYE media at 26 °C for the flagellar expression, Ysp media (TYE media with 0.29 mol/L of NaCl) at 26 °C [17] for the Ysa secretion, and Yop media (91 mL of TYE media, 0.8 mL of 2 M MgCl_2_, and 8 mL of 0.2 M sodium oxalate) at 37 °C [13] for the Ysa secretion. Nalidixic acid (20 µg/mL; SKU: N8878), tetracycline (7.5 µg/mL; SKU: T3383), and streptomycin (30 µg/mL; SKU: S9137) were purchased from Sigma-Aldrich (Merck Korea, Seoul, Republic of Korea). Trichloroacetic acid (Catalog Number: 204-02405) was purchased from FUJIFILM Wako Pure Chemical Co. (Osaka, Japan).

### 2.2. Analysis of T3SS-Secreted Proteins

*Yersinia enterocolitica* cells collected from the precultures grown overnight at 26 °C in TYE media. For Fop secretion, *Y. enterocolitica* was cultured again at 26 °C in TYE medium overnight as the main culture, and cell growth was measured as the absorbance at 600 nm (Abs_600_). For Ysp secretion, precultured cells were inoculated in Ysp media at an Abs_600_ of 0.1, and then the media were incubated at 26 °C for 6 h and the Abs_600_ was measured to determine cell growth. For Yops secretion, precultured cells were inoculated in Yop media at an Abs_600_ of 0.1, and then the media were incubated at 37 °C for 6 h and the Abs_600_ was measured to determine cell growth.

To analyze the proteins secreted via all three T3SSs, cells were removed from the culture media by centrifugation at 7280× *g* and 4 °C for 10 min. One mL of cold, cell-free supernatant was mixed with 0.1 mL of cold trichloroacetic acid solution (500 g of trichloroacetic acid [SKU: T9159; Merck Korea, Seoul, Republic of Korea] in 227 g of water). The mixtures were incubated at 4 °C overnight. After centrifugation at 7280× *g* and 4 °C for 20 min, the liquid supernatant was discarded, and the pellet was resuspended in 1 mL of cold acetone and incubated at 4 °C for 20 min. The acetone was then removed by centrifugation at 7280× *g* and 4 °C for 20 min, and the pelleted protein was resuspended in sodium dodecyl sulfate-polyacrylamide gel electrophoresis (SDS-PAGE) sample buffer (SKU: 70607; Merck Korea, Seoul, Republic of Korea). The volume of SDS-PAGE sample buffer used was proportional to the amount of pellet obtained from a culture with a cell density of Abs_600_ = 1, corresponding to 20 μL. The resuspended Fops and Yops samples were heated to 95 °C for 5 min. Fops and Ysps were separated using SDS-PAGE with a 10% polyacrylamide gel, while Yops were separated using SDS-PAGE with a 12% polyacrylamide gel. After separation, the proteins were visualized through Coomassie blue staining.

### 2.3. YplA Phospholipase Activity Assay

To assess protein secretion, precultured *Y. enterocolitica* cells were inoculated at an Abs_600_ of 0.1 into 100 µL of TYE medium for Fops, 100 µL of Ysp medium for Ysps, and 200 µL of Yops medium for Yops, in 96-well microplates. The plates were then incubated for 6 h at 26 °C for Fops and Ysps secretion or at 37 °C for Yops secretion, and cell growth was determined using the Abs_600_. For Yops-secretion cultures, the calcium chelating activity of sodium oxalate was deactivated by adding 20 µL of 110 mM CaCl_2_. For all samples, 100 µL of cell-free supernatant was transferred to a new 96-well plate after centrifugation at 4300× *g* for 5 min, and 10 µL of 11 × YplA buffer (1.1 M tris(hydroxymethyl)aminomethane at pH 8.0, 5.5% NaCl, 11% tween 80, 11 mM CaCl_2_, and 2.2% sodium azide) was added [35,36]. After incubation at 26 °C for 3 h, the Abs_595_ was measured using a microplate reader (Opsys MR^TM^, Dynex Technologies Inc., Chantilly, VA, USA).

### 2.4. Preparation of the L. obtusiloba Extract

The stem bark of *L. obtusiloba* was purchased from the Jiwoondang Herbal Medicine Store (Seoul, Republic of Korea) and ground to a particle size of ≤1 mm. A total of 3 kg of the ground stem bark was extracted three times by soaking in 5 L of methanol at 45 °C for 5 h. The extraction solvents were then evaporated using a reduced-pressure rotary evaporator (RV10, IKA^®^ Korea, Ltd., Seoul, Republic of Korea), and the residue was dried at 80 °C for 5 days. All extraction, partitioning, and chromatographic purification procedures were independently performed in triplicate using standardized conditions to assess reproducibility. The yields of the ethyl acetate fractions were consistent across batches (±10%), and representative results are shown.

### 2.5. Isolation of Active Ingredients

The methanol extract (353.5 g) was fractionated using butanol, dichloromethane, ethyl acetate, and water. The ethyl acetate fraction (9.5 g) was separated by silica gel vacuum column chromatography (400 mesh) using a solvent mixture of 83.3% dichloromethane and 16.7% methanol, yielding three fractions (E1-1–E1-3). Of these, E1-2 exhibited strong inhibitory activity against YplA phospholipase. Thus, 2.221 g of E1-2 was further separated into three fractions (E2-1–E2-3) through C18 reverse-phase column chromatography with 11.1% methanol. Among these fractions, E2-2 showed strong inhibitory activity against YplA phospholipase, so 889 mg was purified through C18 reverse-phase column chromatography with 9.1% methanol, and E3-3 (final weight: 185 mg) showed strong inhibitory activity against YplA phospholipase.

The chemical structure of the compound in E3-3 was analyzed using INOVA 300 MHz nuclear magnetic resonance (NMR) spectrometer (Varian, Walnut Creek, CA, USA) and a Micromass ZQ detector (Waters Co., Milford, MA, USA) attached to an Alliance HPLC system (Separation Module 2695, Waters Co., Milford, MA, USA). A single compound was identified as inhibiting T3SS protein secretion using a YplA assay. The chemical shifts are reported in δ (ppm) with coupling constant J (Hz).

### 2.6. Morphological Changes to Macrophage Cells Following Y. enterocolitica Infection

RAW 264.7 macrophage cells were cultured at 37 °C and 5% CO_2_ in Dulbecco’s modified Eagle’s medium (DMEM) supplemented with fetal bovine serum at 10% (*v*/*v*), 100 U/mL of penicillin, and 100 μg/mL of streptomycin. The cells were seeded at 1 × 10^5^ cells/well in 12-well cell culture plates with 1 mL of DMEM/well. Cinnamtannin B1 was solubilized in 0.1% DMSO in DMEM prior to application. This vehicle concentration showed no cytotoxic effects on RAW 264.7 cells. All assays were performed under fully solubilized conditions without visible precipitation up to 200 µM.

For infection, *Y. enterocolitica* strains were precultured in 5 mL of TYE media at 26 °C overnight and subcultured at 1:30 into 1.3 mL of Yops medium with or without 100 µmol/L of cinnamtannin B1, with incubation at 37 °C for 2 h. A subset of RAW 264.7 cells were also treated with 100 µM cinnamtannin B1 at 37 °C for 2 h before infection. The RAW 264.7 cells were washed with phosphate-buffered saline (PBS; catalog number: C-9024; Bioneer Co., Daejeon, Republic of Korea) and infected with *Y. enterocolitica* at a multiplicity of infection (MOI) of 50. After centrifugation at 800× *g* for 5 min, the plates were incubated at 37 °C for 5 h under 5% CO_2_. Morphological changes to the RAW 264.7 cells were observed with an inverted microscope (40×; Nikon Instruments Korea, Seoul, Republic of Korea)

### 2.7. Measuring Cell Cytotoxicity Due to Y. enterocolitica Infection

RAW 264.7 macrophage cells were seeded at 2 × 10^4^ cells/well in 96-well microplates containing 100 µL/well of DMEM. The cells were cultured at 37 °C for 24 h under 5% CO_2_. *Y. enterocolitica* strains were precultured overnight in 5 mL of TYE media at 26 °C and subcultured at 1:30 into 1.3 mL of Yops medium with 0, 25, 50, 100, or 200 µmol/L of cinnamtannin B1, with incubation at 37 °C for 2 h. The RAW 264.7 cells were treated with the same concentrations of cinnamtannin B1 before infection. After incubation, the RAW 264.7 cells were washed with PBS and infected with *Y. enterocolitica* at a MOI of 100. After centrifugation at 800× *g* for 5 min, cells were incubated at 37 °C for 1 h under 5% CO_2_. Then, RAW 264.7 cells were washed with PBS and incubated in 100 µL of DMEM with 50 μg/mL of kanamycin for 19 h. The cells were washed with PBS again, and 100 µL of DMEM with 500 mg/L 3-(4,5-dimethylthiazol-2-yl)-2,5-diphenyltetrazolium bromide (MTT) was added to each well. After 4 h of incubation at 37 °C under 5% CO_2_, the media was removed. Twenty μL of dimethyl sulfoxide (DMSO) and 180 μL of a 0.04 N HCl isopropanol solution was added and mixed by shaking for 5 min. The color intensity of formazan derivative produced by the ensuing reaction was measured at 570 nm using an Opsys MRTM microplate reader (Dynex Technologies) [37].

### 2.8. Gene Expression Analysis

The *Y. enterocolitica* strains were treated with 50 μM cinnamtannin B1 at 37 °C for 3 h in Yops media. The total RNA was isolated using TRI reagent (SKU: T3934, Merck Korea, Seoul, Republic of Korea) according to the manufacturer’s instruction, and DNA was removed by incubating the samples at 37 °C for 15 min with DNase I, amplification grade (Catalog number: 18068015; Thermo Fisher Scientific Inc., Waltham, MA, USA). The RNA was then purified using an RNeasy Mini kit (catalog number: 74104; Qiagen Korea Ltd., Seoul, Republic of Korea) according to the manufacturer’s instructions. One µg of purified RNA was subjected to reverse-transcription using random primers and M-MLV Reverse Transcriptase (catalog number: M2410-100; GenDEPOT, Baker, TX, USA). The thermocycler protocol for reverse transcription included 65 °C for 15 min, 30 °C for 10 min, 37 °C for 2 h, and then 70 °C for 20 min. A reverse-transcription polymerase chain reaction (RT-qPCR) analysis was performed using iTaq Universal SYBR Green Supermix (Bio-Rad Laboratories Inc., Hercules, CA, USA). The primers used and the genes they targeted are detailed in Table 1. The RT-qPCR was performed in triplicate in a MiniOpticon Real-Time PCR System (catalog number: CFB3120EDU, Bio-Rad Laboratories Inc., Hercules, CA, USA) with these thermocycler conditions: pre-denaturation at 95 °C for 3 min, followed by 50 cycles of denaturation at 95 °C for 10 s, annealing at 58 °C for 10 s, and extension at 72 °C for 20 s. Melting curves were analyzed to ensure the amplification produced single peaks, indicating specific product amplification [38]. All RT-qPCR reactions were performed in biological triplicate. Relative gene expression was calculated using the 2^−ΔΔCt^ method, and *p*-values were determined using unpaired Student’s t-tests.

### 2.9. Detection of Poly (ADP-Ribose) Polymerase (PARP) by Immunoblotting

The RAW 264.7 macrophage cells were seeded at 3 × 10^5^ cells/well in 6-well plates and cultured at 37 °C for 24 h under 5% CO_2_. The *Y. enterocolitica* strains were precultured in 5 mL of TYE media at 26 °C overnight. The precultured *Y. enterocolitica* bacteria were subcultured at a 1:30 dilution into 1.3 mL of Yops media with or without 100 µmol/L of cinnamtannin B1 and incubated at 37 °C for 2 h. A subset of the RAW 264.7 cells was also pre-treated with 100 µM cinnamtannin B1. The RAW 264.7 cells were then washed with PBS and infected with *Y. enterocolitica* at a MOI of 50. After centrifugation at 800× *g* for 5 min, the cultures were incubated for 3 h at 37 °C under 5% CO_2_. Then, the RAW 264.7 cells were harvested by centrifugation at 4300× *g* for 5 min, washed with 1 mL of PBS, and lysed in cold RIPA buffer (50 mM tris(hydroxymethyl)aminomethane, 150 mM NaCl, 1 mM ethylene-diamine-tetraacetic acid, 1% Triton X-100, 1% deoxycholate, 0.1% sodium dodecyl sulfate, 1 mM dithiothreitol, and 10 μL/mL of a protease inhibitor cocktail). Protein concentrations in the cell lysates were determined using the Bio-Rad Protein Assay Kit II (catalog number: 5000002; Bio-Rad Laboratories Inc., Hercules, CA, USA), and 40 μg of protein was separated using SDS-PAGE with a 12% polyacrylamide gel and transferred to nitrocellulose membranes. The primary antibody, PARP antibody (catalog number: 9542; Cell Signaling Technology, Inc., Danvers, MA, USA), and anti-rabbit secondary antibodies (catalog number: 7074; Cell Signaling Technology, Inc., Danvers, MA, USA) were used at 1:1000 dilutions. The antibodies bound by PARP were visualized using Amersham ECL Detection Reagents (catalog number: RPN2105; Cytiva, Global Life Sciences Solutions Korea Ltd., Incheon, Republic of Korea) according to the manufacturer’s instructions.

## 3. Results

### 3.1. An Ethyl Acetate Fraction of L. obtusiloba Inhibited the Secretion of YplA

**Figure 1:**
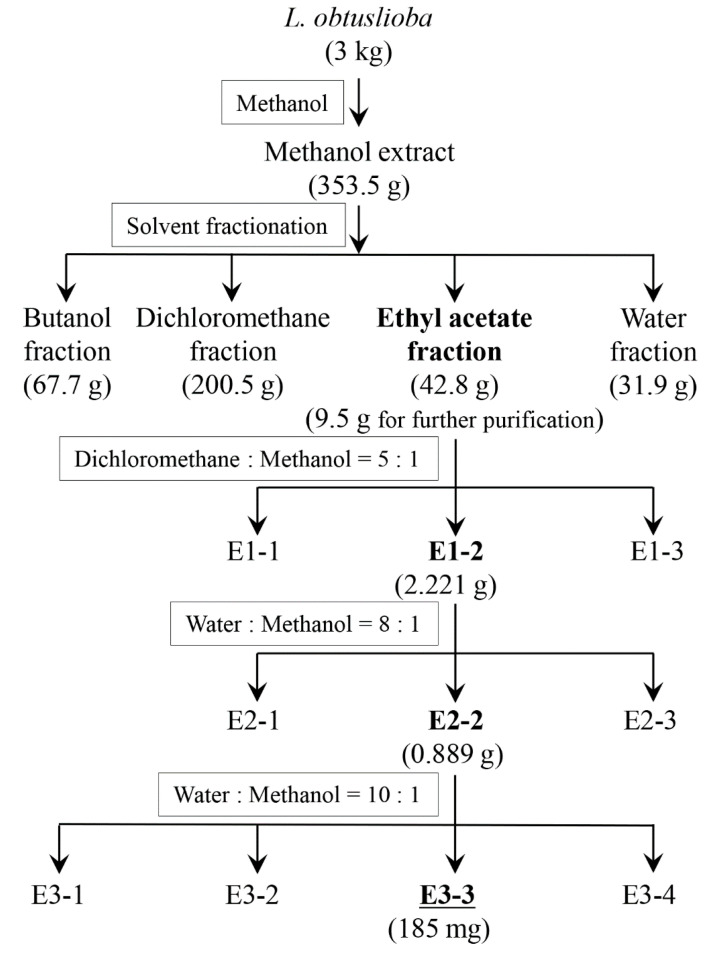
Fractionation procedure for the *L. obtusiloba* extract, with the most active fraction at each step in bold. The separation process was performed to isolate the active compound responsible for the inhibition of YplA phospholipase activity. The amounts of solids obtained at each step are given in parentheses, while the solvent composition used during the separation step is given in the rectangular boxes. All extraction and purification steps were repeated in triplicate, and the active fractions yielded consistent mass and biological activity across batches.

Using methanol, 353.5 g of extract solid was prepared from 3 kg of *L. obtusiloba* bark, representing an 11.43% solid yield (Figure 1). The methanol extract was fractionated using four solvents, butanol, dichloromethane, ethyl acetate, and water, with 67.7 g, 200.5 g, 42.8 g, and 31.9 g of extract solid, respectively.

**Figure 2:**
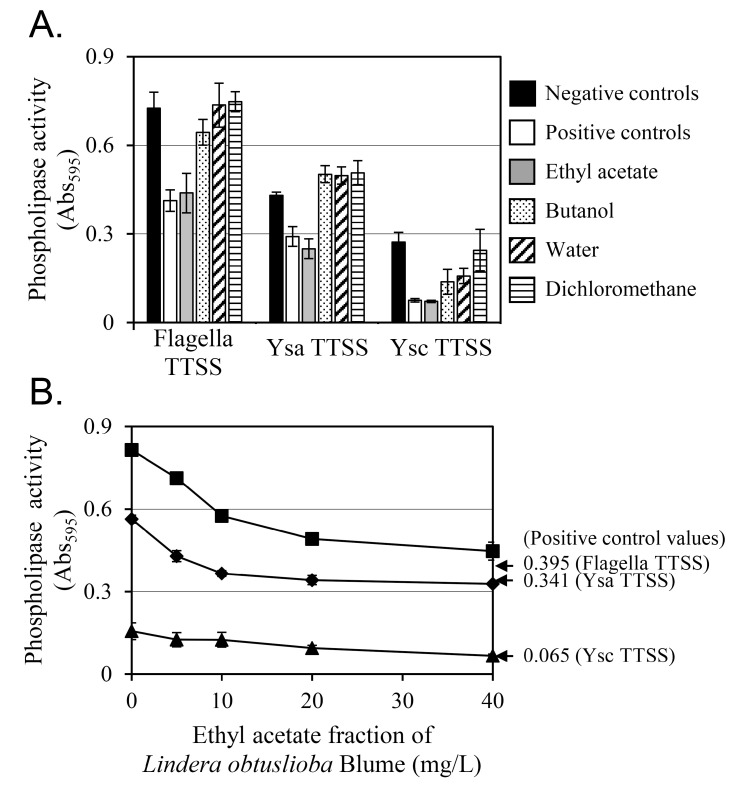
YplA phospholipase activity of *Y. enterocolitica* affected by extracts of *L. obtusiloba*. (**A**) Phospholipase activity was assessed by measuring the turbidity caused by the reaction between calcium and the fatty acids released from Tween 80 hydrolysis by YplA. The negative control (black bars) represents the phospholipase activity of *Y. enterocolitica* KT0004, which expresses YplA. The positive control (white bars) corresponds to *Y. enterocolitica* KT0003, which lacks the YplA overexpression plasmid pGY100. *Yersinia enterocolitica* KT0004 was treated with 100 mg/L of the ethyl acetate (gray bars), butanol (dotted bars), water (diagonally striped bars), and dichloromethane (horizontally striped bars) fractions derived from the methanol extract of *L. obtusiloba*. (**B**) The phospholipase activities of the flagellar (■), Ysa (♦), and Ysc (▲) T3SSs after treatment with the ethyl acetate fraction of *L. obtusiloba* methanol extract at various concentrations. Arrows and values to the right of the graph show the Abs_595_ values for each T3SS from the positive control strain KT0003. Results are presented as the mean ± the standard deviation (error bars) from triplicate experiments.

In a previous study, YplA, a phospholipase, was secreted by *Y. enterocolitica* via three T3SSs—the flagellar, Ysa, and Ysc [12]. The phospholipase activity of the YplA secreted by these three T3SSs was measured along with the change in phospholipase activity induced by 100 µg/mL of extract (Figure 2A). The virulence factor YplA hydrolyzes the ester linkage of Tween 80, releasing a fatty acid. The free fatty acid then reacts with calcium ions, increasing the turbidity of the reaction solution. Thus, the amount of YplA secreted via the T3SS can be quantified by phospholipase activity. The negative control in Figure 2A represents the phospholipase activity of strain KT0004, which overexpressed YplA without any inhibition. The positive control in Figure 2A corresponds to the phospholipase activity of strain KT0003, which lacked the YplA overexpression plasmid, pGY100.

The ethyl acetate fraction (gray bars in Figure 2A) produced the greatest reduction in phospholipase activity, which reached levels very similar to those of the positive control. In contrast, the dichloromethane fraction (horizontal-lined bars in Figure 2A) did not reduce phospholipase activity at all. For the butanol (dotted bars in Figure 2A) and water (diagonal-lined bars in Figure 2A) fractions, a partial reduction in phospholipase activity was observed only in the Ysc T3SS. The ethyl acetate fraction was selected for further study because it exhibited the greatest inhibition.

The phospholipase activity decreased proportionally with increasing concentrations of the ethyl acetate fraction (Figure 2B). At 0 mg/L (the negative control, strain KT0004 with no fraction treatment), the flagellar T3SS showed the highest phospholipase activity, with the Ysc T3SS showing the lowest activity among the three T3SSs. At 40 mg/L of ethyl acetate fraction, phospholipase activity was reduced by 45%, 42%, and 57% for the flagellar, Ysa, and Ysc T3SSs, respectively. For comparison, the positive control (strain KT0003, lacking plasmid pGY100) showed 52%, 39%, and 58% lower phospholipase activity than the negative control (strain KT0004 with 0 mg/L ethyl acetate fraction) for the flagellar, Ysa, and Ysc T3SSs, respectively. These results indicate that YplA-induced phospholipase activity inhibition was greatest at the greatest concentration of the ethyl acetate-soluble fraction of *L. obtusiloba* extract, 40 mg/L.

### 3.2. Cinnamtannin B1 Was Identified in the Ethyl Acetate Fraction of the L. obtusiloba Extract, Which Inhibited YplA Phospholipase Activity Secreted by T3SSs in Y. enterocolitica

The ethyl acetate fraction (9.5 g) was further fractionated to identify the active compound (Figure 1). This fraction had been separated by silica gel (400 mesh) vacuum column chromatography using a solvent mixture of 83.3% dichloromethane and 16.7% methanol, resulting in three fractions: E1-1–E1-3. Among them, E1-2 showed strong phospholipase inhibitory activity. Using C18 reverse phase column chromatography with 11.1% methanol as the eluent, 2.221 g of E1-2 was further fractionated into three subfractions: E2-1–E2-3. Subfraction E2-2 retained strong phospholipase inhibitory activity, and 0.889 g of E2-2 was purified again using C18 reverse phase column chromatography with 9.1% methanol as the eluent. After this, E3-3 showed strong inhibitory activity against YplA phospholipase activity.

The compound from E3-3 was obtained as a brown amorphous powder, and its molecular formula appeared to be C_45_H_36_O_18_ based on the ion peak observed for the [M − H]^−^ ion at m/z 863.2 in the electrospray ionization mass spectrum. The compound’s 1D NMR spectra, including chemical shift (δ, ppm) and coupling constant (J, Hz) values, are presented in Supplementary Table S1 and were interpreted by comparison with values in the literature [39,40] to confirm the structure.

**Figure 3:**
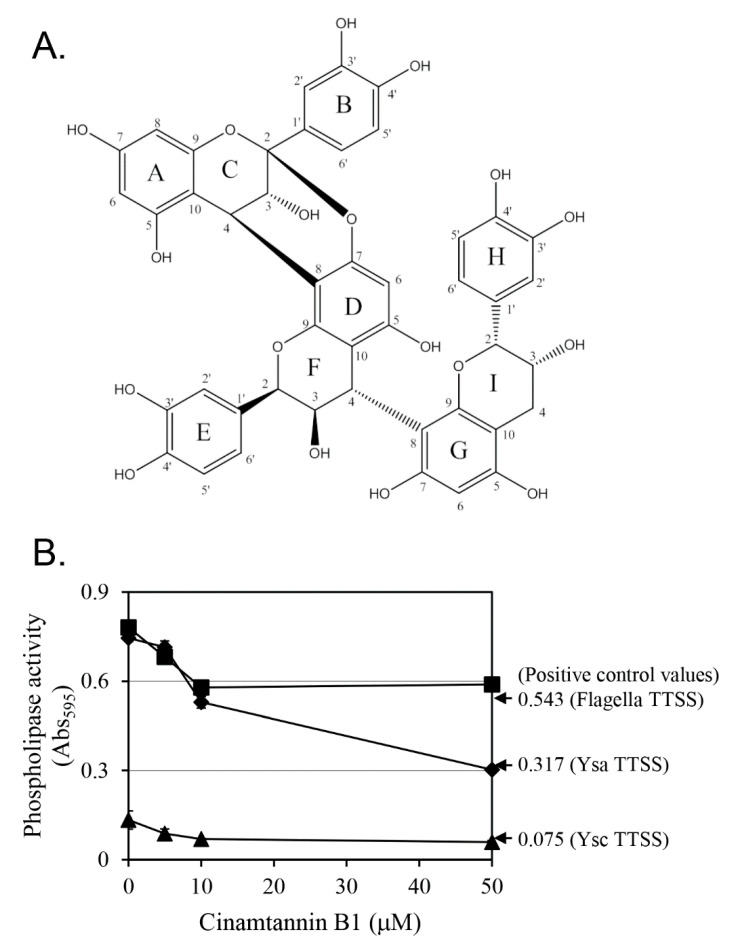
Chemical structure of cinnamtannin B1 (**A**) and analysis of YplA secretion from *Y. enterocolitica* treated with cinnamtannin B1. (**B**) The phospholipase activity of the flagellar (■), Ysa (♦), and Ysc (▲) T3SSs after treatment with cinnamtannin B1 at various concentrations. Results are presented as the mean ± the standard deviation (error bars) of triplicate experiments. Positive control values, shown to the right of the graph, represent the phospholipase activity of strain KT0003.

The ^1^H-NMR spectrum exhibited resonances typical of three ABX systems, indicating the presence of three 1,3,4-trisubstituted aromatic rings (B, E, and H). Additionally, a pair of doublets at δ_H_ 3.46 (d, *J* = 3.7 Hz) and δ_H_ 4.00 (d, *J* = 3.7 Hz) were observed, corresponding to H-3 and H-4 of a doubly linked A-type proanthocyanidin. This structural feature was further supported by the ^13^C-NMR spectrum, which displayed a characteristic ketal carbon signal at δ_C_ 98.87, corresponding to the C-2 position of the C ring, which is consistent with a C-2/C-4 linkage between the ABC and DEF moieties. The ^1^H and ^13^C-NMR spectroscopic data (Table S1) indicated that all units were epicatechins. Based on these analytical data, the active compound was suggested to be epicatechin-(2β→O→7, 4β→8)-epicatechin-(4β→8)-epicatechin, also known as cinnamtannin B1 (Figure 3A).

The inhibitory activity of cinnamtannin B1 against the YplA phospholipase secreted by the T3SSs was evaluated (Figure 3B). For flagellar and Ysc T3SSs, the inhibitory activity was dose-dependent up to 10 µM, and the phospholipase activity at 10 µM cinnamtannin B1 was comparable to that of the positive control. However, for the Ysa T3SS, though a dose-dependent inhibition pattern up to 10 µM was clear, a further reduction was seen at 50 µM. These results suggest that cinnamtannin B1 effectively inhibits YplA secretion mediated by all three T3SSs, with potent activity at a 10 µM concentration.

### 3.3. Protein Secretion via the T3SS Is Inhibited by Cinnamtannin B1

**Figure 4:**
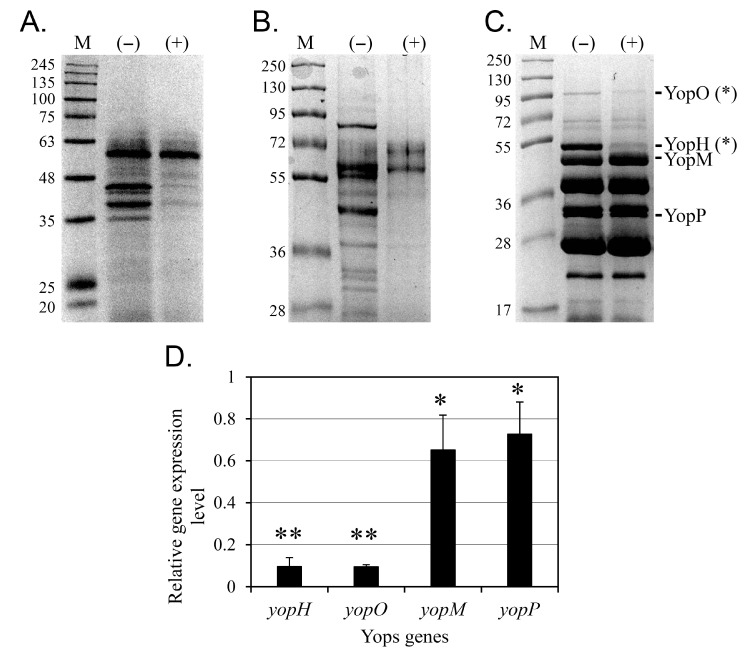
Secreted extracellular proteins and gene expression in *Y. enterocolitica* KT0003 treated with 50 µM cinnamtannin B1. Extracellular protein secretion was analyzed under conditions inducing different T3SSs using SDS-PAGE. Fops (**A**) and Ysps (**B**) were separated on 10% polyacrylamide gels, while Yops (**C**) were separated on a 12% polyacrylamide gel, and all were visualized using Coomassie blue staining. Protein standards (lanes “M”) are shown on the left and labeled with molecular weights (kDa). Untreated (lanes “(−)”) and 50 µM cinnamtannin B1-treated (lanes “(+)”) samples were compared. Cells were grown at 26 °C in TYE medium to induce Fop production (**A**), at 26 °C in Ysp medium to induce Ysps production (**B**), and at 37 °C in Yops medium to induce Yops production (**C**). (**D**) RT-qPCR analysis of *yopH*, *yopO*, *yopM*, and *yopP* expression in *Y. enterocolitica* KT0003 treated with 50 μM cinnamtannin B1. Data represent mean ± SD from three biological replicates, normalized to *gapA* using the ΔΔCt method. Statistical significance is indicated by single asterisk (*) for *p* < 0.05 and double asterisks (**) for *p* < 0.01.

The inhibitory effect of 50 µM cinnamtannin B1 on protein secretion via the three T3SSs was confirmed by SDS-PAGE (Figure 4). Protein secretion in *Y. enterocolitica* KT0003 through the flagellar (Figure 4A) and Ysa (Figure 4B) T3SSs was significantly reduced, with only a few prominent protein bands remaining on the Western blot membranes. For the Ysc T3SS, densitometry was performed to evaluate the secretion of Yop proteins using ImageJ ver 1.54p. Relative intensities of YopH and YopO bands showed statistically significant reduction upon cinnamtannin B1 treatment (*p* < 0.05), while other Yops exhibited no significant changes (Figure 4C). These results suggest that, overall, cinnamtannin B1 effectively inhibits T3SS-mediated protein secretion in *Y. enterocolitica*.

To further investigate the selective effect of 50 μM cinnamtannin B1 on the secretion of YopH and YopO, the expression levels of the genes corresponding to four Yop proteins were analyzed using RT-qPCR (Figure 4D). Gene expression was normalized to the housekeeping gene *gapA* [41]. The results showed that cinnamtannin B1 reduced the expression levels of *yopH* and *yopO* by more than 90%, as quantified by RT-qPCR across three biological replicates (*p* < 0.01). In contrast, the expression levels of *yopM* and *yopP* were moderately reduced by 35% and 27%, respectively (*p* < 0.05). These results suggest that cinnamtannin B1 selectively and strongly represses the gene expression of *yopH* and *yopO*.

### 3.4. Cinnamtannin B1 Prevents the Killing of RAW 264.7 Macrophages by Y. enterocolitica

**Figure 5:**
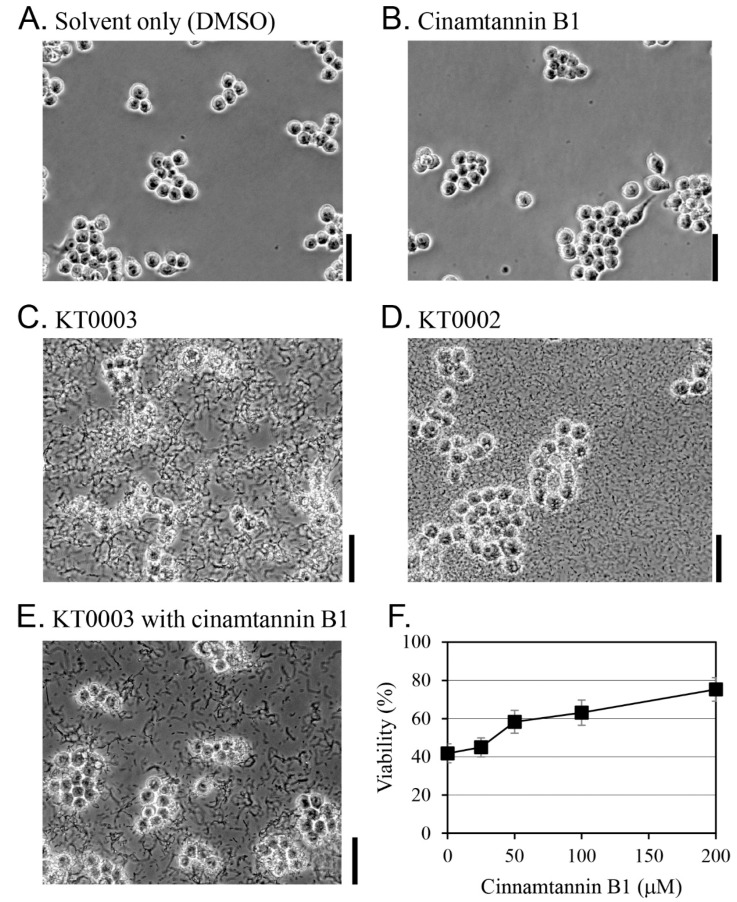
Morphological changes in RAW 264.7 macrophages after infection with *Y. enterocolitica*. Representative images showing RAW 264.7 cells treated with DMSO (**A**), treated with 100 μM cinnamtannin B1 (**B**), exposed to *Y. enterocolitica* KT0003 (**C**), exposed to *Y. enterocolitica* KT0002 (**D**), and exposed to *Y. enterocolitica* KT0003 and treated with 100 μM cinnamtannin B1 (**E**). Cells were observed at 40× magnification, 5 h after exposure. The scale bar to the right of each panel represents 50 μm. (**F**) The viability of RAW 264.7 cells infected with *Y. enterocolitica* KT0003 and treated with cinnamtannin B1, evaluated using the MTT assay. Results are expressed as the mean ± the standard deviation (error bars) of triplicate experiments.

The effect of cinnamtannin B1 on the survival of RAW 264.7 macrophages during *Y. enterocolitica* infection was evaluated through morphological observations and cell viability assays (Figure 5). At concentrations up to 200 μM, cinnamtannin B1 showed no cytotoxicity against RAW 264.7 cells, as evidenced by unchanged cell morphologies at 100 μM cinnamtannin B1 (Figure 5B,E).

Untreated RAW 264.7 cells (Figure 5A) and those treated with 100 μM cinnamtannin B1 alone (Figure 5B) showed no morphological changes. In contrast, cells infected with the virulent *Y. enterocolitica* strain KT0003 showed significant shape disruption (Figure 5C). When infected with the avirulent strain KT0002, RAW 264.7 cells survived and retained normal morphologies despite high bacterial loads (Figure 5D). Notably, under co-treatment with KT0003 and 100 μM cinnamtannin B1, RAW 264.7 cells preserved their shape and viability (Figure 5E), similar to the effect observed with strain KT0002 (Figure 5D).

An MTT assay was performed to quantify cell survival (Figure 5F). In the absence of cinnamtannin B1, strain KT0003 reduced the survival rate of RAW 264.7 cells to 42%. However, cell survival increased to 75% when 200 μM cinnamtannin B1 was added to the medium. These results indicate that cinnamtannin B1 inhibits the virulence of *Y. enterocolitica* KT0003 and protects RAW 264.7 cells from infection in a dose-dependent manner, probably by suppressing the expression of virulence genes (e.g., *yopH* and *yopO*). At the tested concentration (200 µM), cinnamtannin B1 was solubilized using 0.1% DMSO without cytotoxicity, and no precipitation was observed during incubation.

### 3.5. Cinnamtannin B1 Inhibited the Y. enterocolitica-Induced Apoptosis of RAW 264.7 Macrophages

Cinnamtannin B1 significantly increased the cell survival of RAW 264.7 macrophages under attack from *Y. enterocolitica* KT0003 (Figure 5), suggesting reduced apoptosis. Apoptosis was evaluated by analyzing the cleavage of PARP, a key marker of cell apoptosis. Intact PARP is associated with viable cells, whereas cleaved PARP indicates apoptosis.

**Figure 6:**
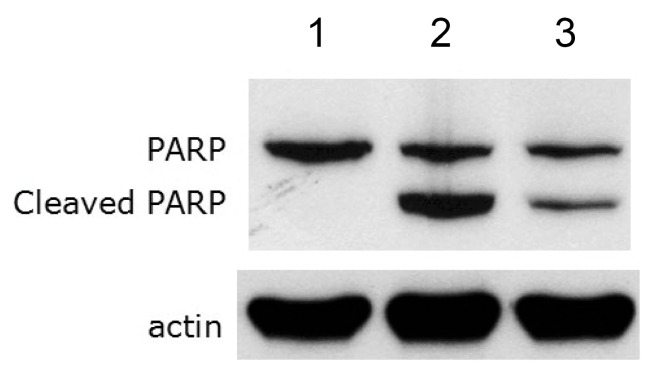
Composite image of the membrane from a Western blot analysis of PARP cleavage in RAW 264.7 macrophages infected with *Y. enterocolitica*. Lane 1 shows the immunoblots from RAW 264.7 cells infected with *Y. enterocolitica* KT0002, which lacks the virulence plasmid. Lane 2 shows the immunoblots from RAW 264.7 cells infected with *Y. enterocolitica* KT0003, which contains the virulence plasmid. Lane 3 shows the immunoblots from RAW 264.7 cells infected with *Y. enterocolitica* KT0003 and treated with 100 µM cinnamtannin B1. The actin immunoblots, used as a control, are shown at the bottom.

Macrophages exposed to *Y. enterocolitica* KT0002, a cured strain lacking the virulence plasmid pYV100, showed intact PARP without cleavage, confirming cell viability (lane 1, Figure 6). In contrast, macrophages exposed to *Y. enterocolitica* KT0003, which harbors the virulence plasmid pYV100, showed significant PARP cleavage, indicating apoptosis (lane 2, Fig, 6), while macrophages exposed to both *Y. enterocolitica* KT0003 and 100 μM cinnamtannin B1 showed significantly reduced PARP cleavage (lane 3, Figure 6). These results confirmed that cinnamtannin B1 effectively inhibited *Y. enterocolitica*-induced apoptosis in RAW 264.7 macrophages, probably by suppressing virulence factors. While PARP cleavage is a widely accepted as a marker of apoptosis, further validation using complementary assays (e.g., caspase-3 or Annexin V) is necessary to confirm these findings at multiple apoptotic checkpoints.

## 4. Discussion

The emergence of antibiotic-resistant bacteria is a major challenge in the treatment of bacterial infections. One promising strategy to address this problem is to target and inactivate bacterial virulence factors—key elements that allow pathogens to infect host cells. Rather than killing the bacteria directly, this approach neutralizes their infection ability, preventing disease progression. By rendering pathogens non-pathogenic, the host’s innate immune system can naturally clear the infection. This strategy provides a viable solution for treating bacterial infections, including those caused by multidrug-resistant strains, where conventional treatments are ineffective.

In this study, we investigated compounds that selectively inactivate the T3SS, using *Y. enterocolitica* as a model organism. The T3SS facilitates the secretion of effector proteins and is a critical virulence factor in animal infections. In *Y. enterocolitica*, YplA, a phospholipase, is secreted under conditions that activate three different T3SSs [12]. In the current study, we showed that the phospholipase activity of secreted YplA serves as a reliable marker for assessing T3SS-mediated protein secretion, especially in high-throughput screenings.

This study showed that cinnamtannin B1, a compound identified from the ethyl acetate fraction of *L. obtusiloba* extract, exhibited potent anti-virulence effects. Specifically, cinnamtannin B1 effectively inhibited the secretion of most proteins associated with both the flagellar and the Ysa T3SSs. However, in the Ysc T3SS, it selectively inhibited the secretion of YopH and YopO, with minimal effect on the secretion of other Yops. While broad T3SS inhibition has been the focus of many polyphenol-based studies, our data suggest that cinnamtannin B1 exerts a more refined mechanism, selectively suppressing *yopH* and *yopO*. These genes encode effector proteins crucial for disabling macrophage cytoskeletal integrity and innate immune responses. RT-qPCR and SDS-PAGE data together indicate a transcriptional-level inhibition, although the specific regulatory pathways (e.g., Ysc master regulators or environmental sensors) remain to be elucidated. This specificity distinguishes cinnamtannin B1 from compounds such as EGCG, which inhibit T3SS broadly without effector discrimination [42]. Further studies will identify the upstream targets responsible for this selectivity.

Yops play a critical role in evading the host’s innate immune response. These proteins interfere with phagocytosis and disrupt host cell signaling [43,44]. Among them, four proteins—YopE, YopH, YopO, and YopT—target the host’s actin cytoskeleton. YopH, a 51 kDa tyrosine phosphatase, is particularly notable for its role in dephosphorylating host cell proteins, disrupting the cytoskeleton, and impairing macrophage phagocytosis [45]. Upon infection, macrophages initiate an oxidative burst and produce nitric oxide through the PI 3-kinase pathway [46]. YopH counteracts these early host defenses by suppressing the production of pro-inflammatory cytokines such as IL-1β and TNFα [47].

YopO, also known as YpkA, is an 81 kDa serine/threonine kinase with structural and functional similarities to eukaryotic serine/threonine kinases [48]. This effector protein contains several functional domains, including a guanine nucleotide dissociation inhibitor (GDI)-like domain, an N-terminal membrane localization domain, and a serine/threonine kinase domain. Together, these enable YopO to inhibit phagocytosis by disrupting Gαq signaling pathways [49]. YopO exerts its effects by recruiting and phosphorylating the host’s actin regulatory proteins, thereby disrupting actin filament regulation. This disruption compromises host cells’ cytoskeletal dynamics, effectively neutralizing macrophage responses and impairing the innate immune defense against bacterial infection [50,51]. By selectively inhibiting YopH and YopO secretion, cinnamtannin B1 demonstrates potential to neutralize key virulence mechanisms, thereby enhancing the host’s ability to fight *Yersinia* infections.

Its inhibition of the effector proteins YopH and YopO, both of which interfere with phagocytic mechanisms, suggests that cinnamtannin B1 has a protective function in preventing *Y. enterocolitica*-induced macrophage death. This cytoprotective function was further supported by a significant reduction in PARP degradation, a key marker of apoptosis. In contrast to previous studies, which have mainly focused on broad-spectrum T3SS inhibitors, this study demonstrates the benefits of the strong selective inhibition of YopH and YopO expression by cinnamtannin B1, providing a new perspective on treatments targeting T3SS-associated pathogenicity.

This finding also highlights YopH and YopO’s critical roles in the infection of host cells. In addition, the targeted inhibition of virulence factors by cinnamtannin B1 minimizes disruption of the host’s resident microbiota, providing a targeted therapeutic strategy. By attenuating bacterial virulence without directly killing the pathogen, cinnamtannin B1 also reduces the risk of antibiotic resistance emergence, providing an alternative candidate to conventional antibiotics. Future studies will be essential to identify the molecular targets of cinnamtannin B1 responsible for the suppression of *yopH* and *yopO* expression, paving the way for the development of novel anti-virulence therapies. Importantly, the selective targeting of YopH and YopO, without broad cytotoxic effects, underlines the therapeutic potential of cinnamtannin B1 as a compound with selective anti-virulence activity.

The potential applications of cinnamtannin B1 as an anti-virulence agent are vast. In addition to treating infections caused by *Y. enterocolitica*, cinnamtannin B1 could be used against a broad range of T3SS-dependent pathogens. Its ability to protect host cells from apoptosis holds great promise for enhancing therapeutic strategies. Additionally, as a plant-derived compound from *L. obtusiloba*, cinnamtannin B1 offers an attractive natural alternative to synthetic antibiotics. Epigallocatechin gallate, a derivative of epicatechin, the core chemical structure of cinnamtannin B1, has been reported to inhibit T3SS function [42]. Similarly, this study shows that cinnamtannin B1 effectively inhibits the flagellar and Ysa T3SSs. Notably, for the Ysc T3SS, which is critical for host cell infection, cinnamtannin B1 exhibits a unique mechanism: the selective suppression of the effector genes *yopH* and *yopO*, rather than a broad-spectrum inhibition of the T3SS’s secretory function.

While our data clearly demonstrate that cinnamtannin B1 reduces *Yersinia*-induced apoptosis—as evidenced by PARP cleavage, cell viability assays, and morphological observations—the inclusion of additional complementary apoptosis markers, such as caspase-3 activation, Annexin V/PI staining, or TUNEL assays, would further strengthen our conclusions. These assays will be prioritized in future in vivo and in vitro studies to more comprehensively validate the anti-apoptotic effects of cinnamtannin B1.

The targets and biological mechanisms underpinning this selective inhibition remain unclear. Future studies addressing these aspects may lead to innovative therapeutic approaches to combating T3SS-dependent pathogens. Furthermore, as this study is primarily based on in vitro experiments, and thus, its findings need to be validated in animal models to evaluate the therapeutic potential of cinnamtannin B1 in more complex biological systems. In addition, the long-term effects on bacterial resistance development and the safety profile of the compound across various cell types need to be thoroughly investigated.

While our study demonstrates the anti-virulence effects of cinnamtannin B1 in vitro, its translational feasibility in vivo remains to be determined. Like many proanthocyanidins, cinnamtannin B1 exhibits low aqueous solubility and limited oral bioavailability, which may constrain its systemic therapeutic applications. Nonetheless, our assay conditions used 0.1% DMSO to enhance solubility without observed cytotoxicity. Future studies should focus on formulation strategies, such as nanoparticle encapsulation or the use of structural analogs, to improve cinnamtannin B1’s pharmacokinetic properties. Additionally, although the MOI values used in our macrophage assays (50–100) are standard for in vitro infection models, they exceed physiological relevant exposure levels. Future studies will therefore aim to evaluate efficacy at lower MOIs and in appropriate in vivo models to more accurately determine the therapeutic potential of cinnamtannin B1 under physiologically relevant conditions.

## 5. Conclusions

Cinnamtannin B1, a compound isolated from *L. obtusiloba*, effectively inhibits the secretion of the key virulence factors YopH and YopO in *Y. enterocolitica*. This selective inhibition disrupts *Y. enterocolitica*’s pathogenicity, increases macrophage viability, and reduces apoptosis, highlighting cinnamtannin B1’s potential as an anti-virulence agent. By targeting the T3SS without directly killing bacteria, cinnamtannin B1 offers a potential strategy to combat bacterial infections while reducing the risk of antibiotic resistance development. Furthermore, given its plant-derived origin, cinnamtannin B1 offers a sustainable and biologically relevant alternative to synthetic antibiotics. Future research on cinnamtannin B1 should focus on in vivo validation, elucidation of the precise molecular mechanisms behind its anti-virulence, and exploration of its synergistic potential when combined with conventional antibiotics. Overall, cinnamtannin B1 and *L. obtusiloba* extracts show great promise for the development of novel anti-pathogenic therapies.

## Figures and Tables

**Table 1:** Primers used in this study.

Target Gene	Primer Direction	Sequence (5′ to 3′)
*gapA* (The housekeeping gene)	Sense	CATCCCAGAACATCATCCC
	Antisense	GCAGTCAGGTCAACAACT
*yopH*	Sense	CTAACTCAAGAAGATACCGCTAA
	Antisense	CTATTACCATTGCCGACACT
*yopM*	Sense	CTTCTTGACTGCGATTTATGC
	Antisense	AACTCTGCGGTAATTCTGG
*yopO*	Sense	ATTCCAACGAAGCCAGAC
	Antisense	GTTATCCGCCTTACATCAGT
*yopP*	Sense	GAGAGAGATAGCCTGTTGAAAA
	Antisense	ACTTATTGTGGGGTAAAGGATTT

## Data Availability

The original contributions presented in this study are included in the article. Further inquiries can be directed to the corresponding author.

## Supplementary Materials

The following supporting information can be downloaded at: https://www.mdpi.com/article/10.3390/pharmaceutics17091217/s1, Table S1: ^1^H (300 MHz) and ^13^C (75 MHz) NMR spectroscopic data for the compound isolated from the E3-3 (methanol-d_4_) subfraction of the *Lindera obtusiloba* extract.

## Abbreviations

The following abbreviations are used in this manuscript:

ATCC	American Type Culture Collection
DMEM	Dulbecco’s modified Eagle’s medium
DMSO	Dimethyl sulfoxide
Fops	Flagellar outer proteins
GDI	Guanine nucleotide dissociation inhibitor
KCCM	Korean Culture Center of Microorganisms
MOI	Multiplicity of infection
MTT	3-(4,5-dimethylthiazol-2-yl)-2,5-diphenyltetrazolium bromide
NMR	Nuclear magnetic resonance
PARP	Poly (ADP-ribose) polymerase
PBS	Phosphate-buffered saline
RT-qPCR	Reverse-transcription polymerase chain reaction
SDS-PAGE	Sodium dodecyl sulfate-polyacrylamide gel electrophoresis
SKU	Stock keeping unit
T3SS	Type III secretion system
Ysps	*Yersinia* secreted proteins
Yops	*Yersinia* outer proteins
