# Supplementary material for: Selective Inhibition of Yersinia enterocolitica Type III Secretion by Lindera obtusiloba Extract and Cinnamtannin B1
Source: Pharmaceutics. 2025 Sep 18;17(9):1217. doi: 10.3390/pharmaceutics17091217 (PMC12473489; doi:10.3390/pharmaceutics17091217)
Supplement: Supplementary file 1 [file pharmaceutics-17-01217-s001.zip › pharmaceutics-3864872-supplementary.pdf]

pharmaceutics

Supplementary Material

Selective Inhibition of *Yersinia enterocolitica* Type III Secretion by *Lindera obtusiloba* Extract and Cinnamtannin B1

Jin-Hee Yoo and Tae-Jong Kim *

Department of Forest Products and Biotechnology, Kookmin University, 77 Jeongneungro, Seongbukgu, Seoul 02707, Republic of Korea; jhy0730@kookmin.ac.kr
* Correspondence: bigbell@kookmin.ac.kr

Academic Editor: Emad L. Izake

Received: 25 August 2025
Revised: 15 September 2025
Accepted: 17 September 2025
Published: 18 September 2025

Citation: Yoo, J.-H.; Kim, T.-J. Selective Inhibition of *Yersinia enterocolitica* Type III Secretion by *Lindera obtusiloba* Extract and Cinnamtannin B1. *Pharmaceutics* **2025**, *17*, 1217. https://doi.org/10.3390/pharmaceutics17091217

Copyright: © 2025 by the authors. Submitted for possible open access publication under the terms and conditions of the Creative Commons Attribution (CC BY) license (https://creativecommons.org/licenses/by/4.0/).

Table S1. H (300 MHz) and ^{13}C (75 MHz) NMR spectroscopic data for the compound isolated from the E3-3 (methanol-d_4) subfraction of the *Lindera obtusiloba* extract.

Ring number	Carbon number	Chemical shift		Ring number	Carbon number	Chemical shift	
		δ_C	δ_H (mult.; *J* in Hz)			δ_C	δ_H (mult.; *J* in Hz)
C	2	98.87		E	1'	130.45	
	3	68.96	3.46 (d; 3.7)		2'	115.36	7.24 (d; 1.8)
	4	27.66	4.00 (d; 3.7)		3'	144.83	
A	5	155.49			4'	145.15	
	6	97.20	5.94 (d; 2.4)		5'	114.67	6.77 (d; 8.1)
	7	156.63			6'	119.91	7.15 (dd; 8.1, 2.1)
	8	95.38	6.01 (d; 2.1)	I	2	82.11	4.06 (s)
	9	153.04			3	66.01	3.94 (s)
	10	103.85			4	29.55	3.05 (m)
B	1'	131.25		G	5	154.70	
	2'	114.65	7.09 (d; 2.1)		6	95.34	6.11 (s)
	3'	144.60			7	154.16	
	4'	145.49			8	107.63	
	5'	116.18	6.85 (d; 9.0)		9	154.40	
	6'	118.85	6.95 (dd; 8.4, 2.1)		10	100.56	
F	2	77.45	5.51 (s)	H	1'	131.53	
	3	71.26	3.97 (s)		2'	114.60	6.77 (d; 1.5)
	4	37.10	4.53 (s)		3'	144.32	
D	5	154.16			4'	144.68	
	6	94.89	5.84 (s)		5'	115.03	6.73 (d; 8.3)
	7	149.86			6'	118.83	6.67 (dd; 8.1, 1.8)
	8	105.05					
	9	150.59					
	10	105.38					
